# Healthcare Professionals’ Acceptance of Digital Cognitive Rehabilitation

**DOI:** 10.3389/fpsyg.2020.617886

**Published:** 2020-11-26

**Authors:** Ineke J. M. van der Ham, Rosalie van der Vaart, Anouk Miedema, Johanna M. A. Visser-Meily, Milan N. A. van der Kuil

**Affiliations:** ^1^Department of Health, Medical, and Neuropsychology, Leiden University, Leiden, Netherlands; ^2^Department of Rehabilitation, Physical Therapy Science and Sports, Brain Center, University Medical Center Utrecht, Utrecht, Netherlands; ^3^Center of Excellence in Rehabilitation Medicine, Brain Center, University Medical Center Utrecht, and De Hoogstraat Rehabilitation, Utrecht, Netherlands

**Keywords:** digital cognitive rehabilitation, technology acceptance, implementation, eHealth, neuropsychology

## Abstract

With technological possibilities in healthcare steadily increasing, more tools for digital cognitive rehabilitation become available. Acceptance of such technological advances is crucial for successful implementation. Therefore, we examined technology acceptance specifically for this form of rehabilitation in a sample of healthcare providers involved in cognitive rehabilitation. An adjusted version of the Technology Acceptance Model (TAM) questionnaire was used, including the subscales for perceived usefulness, perceived ease of use, subjective norm (toward use), and intention to use, which all contribute to actual use of a specific technology. Results indicate a generally favorable attitude toward the use of digital cognitive rehabilitation and positive responses toward the TAM constructs. Only for subjective norm, a neutral mean response was found, indicating that this could pose a potential obstacle toward implementation. Potential differences between subgroups of different age, gender, and professional background were assessed. Age and gender did not affect the attitude toward digital cognitive rehabilitation. Occupational therapists showed lower scores than healthcare psychologists and physiatrists with regard to perceived usefulness, possibly linked to a difference in operational and managerial tasks. The findings of his study stimulate further implementation of digital cognitive rehabilitation, where the role of subjective norms should be specifically considered.

## Introduction

A clear increase in the use of technology in rehabilitation is observable over the last decades. Many of the newly developed methods focus on the rehabilitation of motor skills. For instance, robotics, virtual reality, and advanced motor analyses can be used to improve specific motor activities (e.g., Holden, 2005; Nef and Riener, 2005; Howard, 2017). The effective application of such technology for cognitive rehabilitation is currently less common, but is quickly evolving (see e.g., Mantovani et al., 2020). Within cognitive rehabilitation, technology can be applied to both the content and the format of treatment. In terms of content, cognitive exercises can be digitized, for instance (Schatz and Browndyke, 2002), whereas the format can benefit from communication solutions such as audio or video chat functions to provide care remotely (Kampik et al., 2015). The development of digital treatments is benefitting from widely accessible tools such as virtual reality applications. Recent studies demonstrate the value of such treatment approaches (Edwards et al., 2014; Claessen et al., 2016; van der Kuil et al., 2018), especially in terms of ecologically valid and controllable environments. The traditional approach to rehabilitation involves a team of healthcare professional that provide exercises and instructions for everyday cognitive activities. Commonly, pen and paper workbooks are used to instruct patients, monitor progress, and communicate between healthcare professionals. Notable advantages of digitally based treatments as compared to these traditional counterparts include the automatic and secure storage of test data, highly reliable administration of stimuli, improvements in standardization, and the possibility to administer treatments remotely (Schatz and Browndyke, 2002; Edwards et al., 2014; Kampik et al., 2015). Moreover, the current Corona pandemic situation has accelerated the demand and development of these techniques, which allow for online continuation of treatment (e.g., Hosey and Needham, 2020). Despite the fast increase in the popularity of this form of treatment and its obvious advantages, the implementation of digital cognitive rehabilitation can still be challenging due to various obstacles. Furthermore, this form of rehabilitation extends to a range of clinical applications, including treatment of neurodevelopmental disorders (e.g., Yerys et al., 2018; Voss et al., 2019) Lack of endorsement and lack of acceptance for digital treatment methods among health care providers may pose such obstacles, as their attitude clearly plays a crucial role in the adoption process (Chismar and Wiley-Patton, 2002; Mora et al., 2008). One factor that could affect health care provider attitude is a critical evaluation of earlier methods of digital cognitive rehabilitation, which often focus on restoration of isolated cognitive functions. However, newer methods are currently introduced, which use a more holistic approach, aimed at increasing participation and offering blended care (e.g., Van Heugten et al., 2016; Cogollor et al., 2018). Therefore, the current study is aimed at identifying the attitude of healthcare providers toward digital cognitive rehabilitation, in order to gain insight in this important factor for success of implementing digital cognitive rehabilitation techniques and to pinpoint potential obstacles toward its implementation.

The identification of an individual’s attitude toward a specific form of technology can be accomplished by using the “Technology Acceptance Model” (TAM; Davis, 1989). This scale was originally designed to discover the underlying factors causing a negative attitude toward technology. It is based on the notion that the degree of technology acceptance depends on multiple constructs (Davis, 1989). In 2000, the scale has been updated (Venkatesh and Davis, 2000). Subjective norm, perceived usefulness, and perceived ease of use contribute to the intention to use, which ultimately leads to actual use. Additionally, subjective norm, an evaluation of the preferences of an individual’s peers and superiors, is directly related to perceived usefulness (Chismar and Wiley-Patton, 2002; Dalcher and Shine, 2003; Venkatesh et al., 2003; Cheon et al., 2012; Surendran, 2012).

Individual differences might additionally influence the constructs of the TAM and technology use. Demographic information such as gender and age of potential users had an effect on their degree of acceptance (Venkatesh et al., 2003; Abu-Dalbouh, 2013; Gartrell et al., 2015; Khalifa and Alswailem, 2015; Moore et al., 2015; Almeida et al., 2017). Given the specificity of the current focus on cognitive rehabilitation and the incongruence in the literature, gender, age, and professional background will be considered in our examination of the attitude of healthcare providers toward digital cognitive rehabilitation.

In our use of the TAM, the mean ratings across the different subscales were explored to assess the current state of healthcare providers’ attitude toward digital cognitive rehabilitation. Next, individual items of the questionnaire used were studied in order to identify potential obstacles toward technology acceptance and eventually actual system use. In literature, impact of age, gender, and professional background has been found in some but not all cases, therefore, no clear hypotheses can be formulated and an exploratory approach will be used. Lastly, the outcomes of this study will provide information about the current degree of acceptance for digital cognitive treatments among healthcare providers working in the field of cognitive rehabilitation. The degree of acceptance along with the identification of potential obstacles can be consulted in future implementation of such digital treatment solutions.

## Materials and Methods

### Participants

The target population for the questionnaire consisted of healthcare providers administering cognitive treatment to patients suffering from cognitive complaints. This particularly includes healthcare providers not only working in care facilities with a specialization in cognitive rehabilitation, such as neurological rehabilitation centers, but also more general facilities such as hospitals. In order to answer the questionnaire adequately, a fluent understanding of the Dutch language was required. No requirements for participation were made based on gender or age. Participants were selected and contacted by the researchers, through professional networks concerning rehabilitation, relevant professional social media groups, and email to direct professional contacts. Dutch as well as Belgian practitioners took part in the questionnaire. Ethical approval for the study was provided by the local ethical committee.

### Measures

A questionnaire was used to assess the attitude of healthcare providers toward digital cognitive rehabilitation. The questionnaire was designed based on the core constructs of the TAM and TAM2 (Davis, 1989; Venkatesh et al., 2003). This included the subjective norm construct as well as this has been shown to directly predict the intention to use technology. Each construct is measured with a separate subscale. In order to answer the main question of the attitude of healthcare providers toward digital cognitive rehabilitation, we examined the scores of each of the subscales of the TAM2 (perceived usefulness – six items, perceived ease of use – six items, subjective norm – three items, and intention to use – two items; for a complete list of questions, see Table 1). To avoid confusion and to ease comparability to other studies based on the TAM, we defined the constructs in the terms of Venkatesh and Davis (2000). As such, the construct of perceived usefulness was defined as the belief the participant has about the extent the use of the cognitive rehabilitation program will enhance their job performance. The construct of perceived ease of use was defined as the extent to which the participant believes the use of the program will be effortless. The construct of subjective norm was defined as the participants’ impression that the use of the program would be or would not be encouraged by peers or superiors important to the respondent. Intention to use referred to the intention to use the technology, provided it is available.

**Table 1:** List of individual items of the questionnaire with mean scores of all participants grouped together.

Subscale	Item	Mean (*SD*)	*t*
Perceived usefulness	Using digital cognitive treatments would improve the care I provide	4.80 (1.16)	8.42^**^
	Using digital cognitive treatments would increase my productivity	4.54 (1.22)	5.42^**^
	Using digital cognitive treatments would make the care I provide more effective	4.82 (1.18)	8.50^**^
	Using digital cognitive treatments would be useful for my work	4.99 (1.28)	9.37^**^
	Using digital cognitive treatments would enable me to provide care for my patients more quickly	4.72 (1.47)	5.95^**^
	Using digital cognitive treatments would make it easier to provide care for my patients	4.70 (1.32)	6.43^**^
Perceived ease of use	My interaction with digital cognitive treatments would be clear and understandable	4.27 (1.11)	2.89^*^
	Interacting with digital cognitive treatments would not require a lot of effort	4.35 (1.11)	3.87^**^
	I would find digital cognitive treatments easy to use	4.43 (1.08)	4.82^**^
	I would find it easy to apply digital cognitive treatments for what I want them to do	4.08 (1.36)	0.73
	Learning to provide digital cognitive treatments would be easy for me	5.30 (1.11)	14.16^**^
	It would be easy for me to become skillful at using digital cognitive treatments	5.29 (1.17)	13.44^**^
Subjective norm	Most of my patients would welcome me using digital cognitive treatments	4.07 (1.47)	0.56
	My superior(s) think(s) that I should use digital cognitive treatments	4.06 (1.66)	0.45
	Colleagues who are important to me think I should use digital cognitive treatments	3.78 (1.52)	−1.72
Intention to use	If I had access to digital cognitive treatments, I would intend to use them	5.37 (1.36)	13.44^**^
	If I had access to digital cognitive treatments, I predict I would use them	5.37 (1.29)	12.87^**^

Each score was contrasted with the neutral value of 4.0 with a Bonferroni corrected one-sample *t*-test (score range 1–7). *SD* = standard deviation.

* *p* < 0.01;

** *p* < 0.001.

The questionnaire was supplemented by demographic and job related questions to additionally explore the potential impact of age, gender, and professional background on the attitude toward digital cognitive rehabilitation. Questions were selected and rephrased based on relevance to healthcare providers working in the field of cognitive rehabilitation. We expected the Cronbach’s alpha scores of the constructs used in this questionnaire to be similar to the ones found in the originals. This entailed a Cronbach’s alpha score of approximately 0.86–0.98 for the perceived usefulness, 0.79–0.98 for the perceived ease of use, 0.81–0.95 for the subjective norm, and 0.82–0.97 for the intention of use (Davis, 1989; Hu et al., 1999; Venkatesh and Davis, 2000; Chismar and Wiley-Patton, 2002; Liang et al., 2003; Yi et al., 2006; Van Schaik et al., 2010; Asua et al., 2012). The possible professional backgrounds of the participants were grouped into meaningful response options. Five categories were determined based on the most likely options within our target demographic. These job categories were occupational therapist, physiatrist, healthcare psychologist (post-graduate level), psychologist, and cognitive therapist. An additional “other” category was added to make the item exhaustive.

### Procedure

At the beginning of the questionnaire, the participants were given a brief explanation of the purpose of the study. Next, the participants were asked to digitally give their informed consent. First, demographic information including their gender, age, professional background, years as a healthcare professional, years of experience with cognitive rehabilitation, and self-reported internet skills were collected. This was followed by 17 questions to measure the participants’ perceived usefulness, perceived ease of use, their subjective norm, and their intention to use the program. These questions were all measured on a 7-point Likert scale following an item phrased as a statement. The scores ranged from 1 (complete disagreement) to 7 (complete agreement), with 4 as the neutral center of the range. Finally, participants were asked additional questions to indicate their preference for several specific design related aspects of a digital cognitive rehabilitation tool developed by the researchers. These last questions were not part of the current study.

### Statistical Analysis

The program IBM SPSS Statistics version 25 was used to conduct the analyses. Cronbach’s alpha for all subscales was determined by conducting a reliability analysis on all items of the subscale. All mean scores were compared to the neutral center of the response options (4.0) to evaluate whether or not participants significantly showed agreement or disagreement for each subscale, using Bonferroni corrected one-sample *t*-tests. Additionally, the individual scores per item were evaluated in the same way, in order to identify potential specific obstacles to the acceptance and use of digital cognitive treatment. Lastly, for gender, age group, and professional background, the nonparametric Kruskal-Wallis H test was performed to identify potential significant differences between groups. For age, participants were divided into three age groups of similar size: younger (<31), middle (31–40), and older (>40). An alpha below 0.05 was considered significant in all analyses and Bonferroni correction was applied in case of multiple comparisons.

## Results

### Participants

In total, 147 participants completed the questionnaire, with a mean age of 38.2 (*SD* = 10.2, range 22–63). A description of the demographic characteristics and self-reported internet skills of the sample is provided in Table 2. The sample was skewed in terms of gender, had a sufficiently varied age range, and covered all professional groups included. However, there was only one cognitive therapist among the participants; therefore, this individual was grouped with the “other” category. All participants indicated at least an average level of internet skills.

**Table 2:** Demographic variables of the sample.

Variable	Response option	N (%)
Gender	Female	128 (87.1)
	Male	19 (12.9)
Professional background	Occupational therapist	45 (30.6)
	Psychologist	28 (19.0)
	Healthcare psychologist	30 (20.4)
	Physiatrist	24 (16.3)
	Cognitive therapist	1 (0.7)
	Other^*^	32 (21.8)
Years as healthcare worker	1–5 years	35 (23.8)
	6–10 years	35 (23.8)
	11–20 years	50 (34.0)
	>20 years	27 (18.4)
Experience cognitive treatment	1–5 years	62 (42.2)
	6–10 years	48 (32.7)
	11–20 years	30 (20.4)
	>20 years	7 (4.8)
Internet skills	Very poor	0
	Poor	0
	Average	19 (12.9)
	Good	69 (46.9)
	Very good	59 (40.1)

* For example, clinical psychologist, clinical neuropsychologist, and physical therapist.

### Subscale Scores

Table 1 depicts all mean scores for all items included and the outcome of the one-sample *t*-tests, comparing the mean scores to 4.0, the neutral center of the scale used. Table 3 depicts mean scores for each subscale, along with Cronbach’s alpha, and the outcome of the one-sample *t*-tests, comparing the mean scores to 4.0. Results indicate that Cronbach’s alpha was well within the expected ranges for perceived usefulness, perceived ease of use, and intention to use. For all three subscales, the mean score was significantly higher than neutral. Subjective norm, however, showed a lower Cronbach’s alpha than expected and did not significantly differ from neutral. Therefore, it is more appropriate to assess scores for the three individual items rather than the subscale as a whole.

**Table 3:** Mean scores for each of the technology acceptance subscales and for all participants grouped together.

Subscale	N items	Mean (*SD*)	Cronbach’s alpha	*t* (comparison to 4.0)
Perceived usefulness	6	4.76 (1.01)	0.884	9.13^*^
Perceived ease of use	6	4.62 (0.88)	0.851	8.56^*^
Subjective norm	3	4.00 (1.20)	0.664	−0.30
Intention to use	2	5.37 (1.25)	0.975	13.31^*^

Reliability was assessed by calculating Crohnbach’s alpha, and each score was compared to the neutral value of 4.0 with a Bonferroni corrected one-sample *t*-test (score range 1–7). *SD* = standard deviation. Two-tailed, corrected for multiple comparisons (alpha = 0.0125).

* *p* < 0.001.

### Identification of Possible Obstacles

All individual items were included in a two-tailed, one-sample *t*-test, corrected for multiple comparisons (alpha: 0.05/17 = 0.0029; see Table 1). All individual items of the subscales perceived usefulness and intention to use had mean scores significantly above 4.0, the neutral center of the scale. For the perceived ease of use, all individual items were significantly higher than 4.0, with the exception of “I would find it easy to apply digital cognitive treatments for what I want them to do.” This specifies that general use is perceived as eas, with the exception of the application of the treatment in practice. Furthermore, all three items of the subjective norm were not significantly different from 4.0, indicating that the subjective norm as presented by patients, superiors, or colleagues is not favorable.

### Individual Differences

Lastly, the impact of individual differences on the subscale scores was assessed. In Table 4, all means scores per subgroup are provided for each of the four subscales. As gender was skewed, a Mann-Whitney U test was used as a nonparametric alternative. No significant differences between males and females were found (*p* > 0.10 in all cases).

**Table 4:** Mean scores for each subscale divided by the subgroups of the sample, based on gender, age group, and professional background.

Factor	Subgroup	N	Perceived usefulness	Perceived ease of use	Subjective norm	Intention to use
Gender	Males	19	4.74 (1.05)	4.60 (1.00)	4.16 (1.12)	5.66 (0.99)
	Females	128	4.77 (1.01)	4.62 (0.86)	3.94 (1.21)	5.33 (1.28)
Age group	Younger (22–30)	41	4.85 (0.87)	4.83 (0.75)	4.01 (1.19)	5.56 (1.19)
	Middle (31–40)	50	4.64 (1.01)	4.55 (0.91)	3.86 (1.33)	5.24 (1.33)
	Older (41–63)	56	4.81 (1.11)	4.52 (0.92)	4.04 (1.09)	5.35 (1.22)
Professional background	Occupational therapists	45	4.33 (1.12)	4.37 (0.87)	3.61 (1.25)	5.06 (1.46)
	Psychologists	28	4.70 (0.97)	4.64 (0.92)	3.92 (1.27)	5.48 (1.19)
	Healthcare psychologists	30	5.05 (0.84)	4.62 (0.86)	4.06 (1.11)	5.42 (1.21)
	Physiatrists	24	5.06 (0.93)	4.76 (0.86)	4.36 (1.01)	5.52 (0.99)
	Other	20	5.04 (0.87)	5.00 (0.79)	4.25 (1.17)	5.68 (1.09)

Standard deviations in parentheses.

To assess the impact of age, the participants were grouped into three age groups, roughly based on the distribution of participants: younger (22–30), middle (31–40), and older (41–63). A one-way ANOVA on the mean scores of the four subtasks did not reveal any significant differences between the three age groups.

A nonparametric approach was also appropriate for the analysis of different professional categories. An independent samples Kruskal-Wallis test was performed and showed that for perceived ease of use, subjective norm, and intention to use, no significant differences were found between professional categories. In contrast, the scores for perceived usefulness were significantly different between professional categories (*p* = 0.014). A Bonferroni-corrected *post hoc* analysis showed that the scores of the occupational therapists were significantly lower than those of the healthcare psychologists and the physiatrists (*p* < 0.05 in both cases).

## Discussion

There is an ongoing increase in the availability of digital cognitive rehabilitation tools with digital applications both in terms of format and content. Technology acceptance is a key in the successful implementation of such treatment protocols as it has been shown to accurately predict actual system use. Here, we studied technology acceptance among healthcare providers in order to answer the main question, concerning the attitude of healthcare providers toward digital cognitive rehabilitation. First, the mean ratings across the different elements of the TAM were explored. Next, individual items of the questionnaire used were studied in order to identify potential obstacles toward technology acceptance and eventually actual system use. Lastly, the impact of individual characteristics including age, gender, and professional background was examined.

First of all, with regard to digital cognitive rehabilitation, health care providers showed convincing levels of agreement with perceived usefulness, perceived ease of use, and the intention to use. In contrast, for the subjective norm subscale, the mean scores showed that this factor is regarded neutrally by our participants. Furthermore, Cronbach’s alpha was rather low for this particular subscale. Therefore, it is informative to also consider each individual item. This analysis revealed that for all three sources of subjective norm included – patients, superiors, colleagues – a neutral attitude is present. This presents a potential obstacle toward technology acceptance and eventually actual system use and is therefore an important element in the implementation of digital cognitive rehabilitation tools. The interpretation of this effect could be 2-fold: either subjective norm is not as high as it needs to be to stimulate system use or the subjective norm is neutral because the attitude of peers and superiors is not known. In the first case, establishing a more positive attitude toward digital cognitive rehabilitation, established by, e.g., visible use of such technology and exchange of positive experiences, could promote system use. In the latter case, a more explicit discussion of attitude concerning digital cognitive rehabilitation would be appropriate, e.g., by discussion this in formal meetings and with patient organizations (e.g., Ploeg et al., 2007; Andreassen et al., 2015). In line with this finding, it should be noted that only few effective methods are currently in use, due to recent improvements in terms of content and required technology. A number of methods have been available for longer, but have not been able to show clear positive results as they often focus on restoration of isolated cognitive functions. In contrast, newer methods use a more holistic approach, in which participation and blended care are focused on (e.g., Van Heugten et al., 2016; Cogollor et al., 2018). Only a limited number of studies are currently available for effective cognitive digital cognitive rehabilitation due to its novelty and the need of follow-up study (e.g., Larson et al., 2014; Mansbach et al., 2015). The process of creating a positive subjective norm is hindered by the scarcity of successful and commendable methods. Furthermore, there is substantial variation in the application of cognitive rehabilitation, in terms of, e.g., pathology, patient characteristics, and specifications of cognitive deficits. Combined with the observation that scores are especially high for the intention to use items, this suggests that health care providers are highly willing to use effective novel methods for digital cognitive rehabilitation, which are not yet widely available. In line with this, implementation strategies that target subjective norms are recommended, e.g., gradual implementation of novel technology, starts with a small group of enthusiastic users (e.g., De Veer et al., 2011).

In the creation of the TAM2, demographic factors were included, with a direct relationship to perceived ease of use (Venkatesh et al., 2003). However, findings on the impact of these factors have been contradictory. Gender may affect the overall acceptance of technology, with a higher level of acceptance of digital therapeutic tools for males, in comparison to females (Mora et al., 2008). In contrast, Khalifa and Alswailem (2015) found that gender did not have a significant influence on the satisfaction of a system. With regard to age, Mora et al. (2008) report a specific age effect for digital chat sessions replacing tradition face-to-face treatment. Psychologist with an older age was more accepting. Similarly, Gartrell et al. (2015) found that older nurses’ approval of an electronic health record for patients was higher in comparison to younger nurses. However, Schnall and Bakken (2011) found no significant relationship between the age of the user and their acceptance for health information technology. In addition to age and gender, professional background can be of impact in acceptance of healthcare technology. Khalifa and Alswailem (2015) found that especially pharmacists and physicians were less inclined to endorse health information technology, while nurses, technicians and administrators did not differ from one another. Van der Vaart et al. (2016) found that mental health counselors tended to have a higher use as well as intention to use online interventions than primary care psychologists. In contrast, Schnall and Bakken (2011) have found no relationship between the professional backgrounds of several different employment classes working in healthcare. These different professional backgrounds included several management positions, social workers, and case follow-up workers. In short, literature is unclear about the impact of demographic variables; therefore, an examination of individual differences was performed. It should be noted that gender did not affect any of the subscales included. Therefore, gender is not expected to have a substantial contribution to actual system use. Age of the health care provider also did not show any effect on the degree of agreement to any of the four subscale of the TAM. Lastly, professional background affected only perceived usefulness. It was found that occupational therapists responded with less agreement to perceived usefulness, in comparison to healthcare psychologists and physiatrists. In terms of task description, the healthcare psychologists and physiatrists are concerned more with an overview of treatment plans for individual patients and generally more involved with management tasks, where occupational therapists are more hands-on in their daily activities and executing the selected treatment plans.

It should be noted that our sample was of sufficient size to accurately assess technology acceptance at group level, but that the individual characteristics of gender and professional background were rather skewed in the sample. Non-parametric statistics were selected to accommodate the sample composition in the analyses. It should be noted that the current questionnaire was focused on the perspective and the opinions of healthcare providers. Another limitation could be that all questions were phrased positively, which could stimulate more positive responses. However, we aimed to use the TAM in the original format, as this has been validated in a range of studies (Davis, 1989; Venkatesh and Davis, 2000). Other potential threats toward successful implementation like policy, insurance, and financial considerations are not considered, but could have a significant impact as well. This may be a prominent cause of why there is currently no common use of this technology. However, such potential barriers should be surveyed among managers and directors, rather than healthcare providers. Lastly, a potential threat of insufficient computer skills was addressed by verifying the level of internet skills in our sample, and we found that all participants indicated at least average internet skills.

To conclude, technology acceptance for digital cognitive rehabilitation is considerable among a sample of healthcare providers with experience in cognitive rehabilitation. Our findings indicate that one potential obstacle toward technology acceptance and eventually actual systems use lies with the subjective norm as perceived by health care providers. Overall, they consider the norms as implied by patients, superiors, and colleagues as neutral. To reach successful implementation, we advise to specifically address this issue in the implementation process, with, e.g., starting with a small group of enthusiastic users, followed by gradual expansion of use. Lastly, systematic individual variation seems limited, and the age and gender do not appear to have an impact. Only professional background, most likely linked to a difference in focus on execution vs. policy affects perceived usefulness to some extent. Overall, the current results indicate that healthcare professionals hold a positive attitude toward digital cognitive rehabilitation tools. The combination of this receptive attitude, technological advances, and increasing strain on healthcare provide ample opportunities for the development and implementation of evidence-based rehabilitation tools.

## Data Availability Statement

The raw data supporting the conclusions of this article will be made available by the authors, without undue reservation.

## Ethics Statement

The studies involving human participants were reviewed and approved by the Committee of Ethics Psychology, Leiden University. The patients/participants provided their written informed consent to participate in this study.

## Author Contributions

AM, MK, and IH performed data processing and written the manuscript. AM and IH performed data analyses. AV and RV critically revised the manuscript. All authors contributed to the article and approved the submitted version.

### Conflict of Interest

The authors declare that the research was conducted in the absence of any commercial or financial relationships that could be construed as a potential conflict of interest.
